# Association between early preterm birth and maternal exposure to fine particular matter (PM_10_): A nation-wide population-based cohort study using machine learning

**DOI:** 10.1371/journal.pone.0289486

**Published:** 2023-08-07

**Authors:** Eun-Saem Choi, Jue Seong Lee, Yujin Hwang, Kwang-Sig Lee, Ki Hoon Ahn

**Affiliations:** 1 Department of Obstetrics and Gynecology, Korea University College of Medicine, Korea University Anam Hospital, Seoul, Korea; 2 Department of Pediatrics, Korea University College of Medicine, Korea University Anam Hospital, Seoul, Korea; 3 AI Center, Korea University College of Medicine, Korea University Anam Hospital, Seoul, Korea; Affiliated Hospital of Nantong University, CHINA

## Abstract

Although preterm birth (PTB), a birth before 34 weeks of gestation accounts for only less than 3% of total births, it is a critical cause of various perinatal morbidity and mortality. Several studies have been conducted on the association between maternal exposure to PM and PTB, but the results were inconsistent. Moreover, no study has analyzed the risk of PM on PTB among women with cardiovascular diseases, even though those were thought to be highly susceptible to PM considering the cardiovascular effect of PM. Therefore, we aimed to evaluate the effect of PM_10_ on early PTB according to the period of exposure, using machine learning with data from Korea National Health Insurance Service (KNHI) claims. Furthermore, we conducted subgroup analysis to compare the risk of PM on early PTB among pregnant women with cardiovascular diseases and those without. A total of 149,643 primiparous singleton women aged 25 to 40 years who delivered babies in 2017 were included. Random forest feature importance and SHAP (Shapley additive explanations) value were used to identify the effect of PM_10_ on early PTB in comparison with other well-known contributing factors of PTB. AUC and accuracy of PTB prediction model using random forest were 0.9988 and 0.9984, respectively. Maternal exposure to PM_10_ was one of the major predictors of early PTB. PM_10_ concentration of 5 to 7 months before delivery, the first and early second trimester of pregnancy, ranked high in feature importance. SHAP value showed that higher PM_10_ concentrations before 5 to 7 months before delivery were associated with an increased risk of early PTB. The probability of early PTB was increased by 7.73%, 10.58%, or 11.11% if a variable PM_10_ concentration of 5, 6, or 7 months before delivery was included to the prediction model. Furthermore, women with cardiovascular diseases were more susceptible to PM_10_ concentration in terms of risk for early PTB than those without cardiovascular diseases. Maternal exposure to PM_10_ has a strong association with early PTB. In addition, in the context of PTB, pregnant women with cardiovascular diseases are a high-risk group of PM_10_ and the first and early second trimester is a high-risk period of PM_10_.

## Introduction

Preterm birth (PTB), a delivery before 37 ^0/7^ weeks of gestation, has been an unsolved major problem in obstetrics for a long time. PTB is divided into early and late PTB according to gestational age (GA). Early PTB is defined as a delivery occurring before 34 ^0/7^ weeks of gestation and late PTB is defined as a delivery occurring between 34 ^0/7^ and 36 ^6/7^ weeks of gestation [1]. PTB accounts for up to 10% of total global births. Early PTB rate was about 2.8% in 2019 in United States and has not decreased over the past decades [2–5]. Although early PTB rate is relatively lower than late PTB rate, early PTB has more significant clinical impact. The mortality of infants born in early PTB period was more than 5 times higher than that of infants born in late PTB in the United States in 2018 [6]. Moreover, early PTB neonates are also at more risk of various morbidities than late PTB neonates [7]. Major complications of neonates including respiratory distress syndrome (RDS), intraventricular hemorrhage (IVH), and even long-term neurodevelopmental morbidities increase with decreasing GA [8–10]. For these reasons, prediction and management of early PTB have always been important issues.

Various factors associated with PTB ranging from genetic features to environmental factors have been reported [11–14]. Among various environmental factors affecting PTB, air pollution, especially exposure to fine particulate matter (PM), has drawn increasing attention in recent decades. Many studies about the association between PM and PTB was also conducted, but the results were conflicting [12–22]. Huynh et al. have reported that maternal exposure to PM can increase the risk of PTB while Pereira et al. could not find a significant association between the two [15, 16]. Several meta-analyses have been conducted to examine the association between PTB and PM, but their results were also inconsistent [23–26]. Ju L et al demonstrated that the exposure of PM10 throughout pregnancy was associated with the increased risk of moderate PTB (delivery at 32–36 weeks of gestation) with a relative risk (RR) of 1.80 (95% confidence interval [CI]: 1.05–1.11) and very PTB (28–31 weeks of gestation) with a RR of 1.13 (95% CI: 1.06–1.21) [23]. However, Yu Z et al reported no significant association between PM10 and moderate and very PTB [24]. Therefore, the association between PM10 and PTB is not yet definitive.

PM_10_ which is particles with an aerodynamic diameter equal or less than 10 μm is a well-known risk factor for cardiovascular diseases [27–30]. Several previous studies demonstrated that the cardiovascular diseases of pregnant women are associated with the increased risk of PTB [31–34]. Furthermore, the more severe cardiovascular diseases are, the greater the risk of PTB. Based on the association between PM_10_ and cardiovascular diseases, it is postulated that pregnant women with cardiovascular diseases may be more susceptible to the effect of PM_10_ on PTB. However, the a about the effect of PM_10_ on pregnant women with cardiovascular diseases is lacking.

Therefore, this study aimed to evaluate the effect of PM_10_ on early PTB compared with effects of known PTB-contributing factors by establishing a prediction model of early PTB using machine learning. In this study, we used data extracted from Korea National Health Insurance (KNHI) claims and concentration of PM_10_ estimated by the national system. In addition, we compared effects of PM_10_ on PTB in pregnant women with cardiovascular diseases and those without cardiovascular diseases.

## Methods

### Study population

This nation-wide population-based cohort study included women aged 25 to 40 years. Singleton primiparous women who delivered babies in 2017 were included. Those who had late PTB were excluded. Data were extracted from KNHI claims. In South Korea, more than 97% of total population are enrolled in KNHI. The database of KNHI contains almost all data covered by the insurance under the National Health Insurance System. KNHI claims data were provided after de-identification according to the Act on the Protection of Personal Information [35]. This retrospective cohort study was approved by the Institutional Review Board (IRB) of Korea University Anam Hospital on November 5, 2018 (2018AN0365). Informed consent was waived by the IRB.

### Variables

The dependent variable was early PTB in 2017. All variables except for PM_10_ were introduced according to the ICD-10 Code and procedure code (S1 Table). PM_10_ concentration by region was provided by the National Ambient Air Monitoring System in South Korea. The National Ambient Air Monitoring System in South Korea consists of 505 stations covering all 162 cities, countries, and districts in the entire nation. By using the demographic information of the study population that was provided from the KNHIS database, we matched the monthly concentration of PM_10_ to each participant. The missing data of PM10 concentration were imputed using median substitution of the PM10 concentration obtained from a nearby monitoring station. A total of 55 independent variables covered the following information: (1) PM_10_ data in 2016 using regional PM_10_ concentration matched with the residence address of study population, including PM_10_ concentration data of specific month (from January 2016 to December 2016) and PM_10_ concentration of each month before delivery (1 to 10 months before delivery); (2) demographic/socioeconomic determinants in 2017 including age and socioeconomic status measured by an insurance fee with the range of 0 (the lowest group) to 20 (the highest group); (3) obstetric and gynecologic diseases (namely, placenta previa, threatened abortion, incompetent internal os of cervix, gestational diabetes, hypertensive disorders during pregnancy (HDP) including gestational hypertension, preeclampsia and eclampsia, congenital malformation of uterus, pelvic inflammatory disease, vaginitis, endometriosis, abnormal menstruation, recurrent miscarriage or infertility) for any year between 2002 and 2016; (4) cardiovascular diseases (i.e., acyanotic congenital heart diseases (CHD), cyanotic CHD, arrhythmia, cardiomyopathy, congestive heart failure (CHF), ischemic heart disease (IHD), and cardiac arrest) for any year between 2002 and 2016; (5) other medical diseases, including hypertension, diabetes, hyperlipidemia, anemia, pulmonary embolism, sepsis, and stroke; and (6) medication history (that is, benzodiazepine, calcium channel blocker (CCB), nitrate, progesterone, hypnotic/sedative drug (antihistamine, zolpidem, eszopiclone, pentobarbital sodium, and benzodiazepine derivates), and tricyclic antidepressant (TCA)) in 2002–2016. Women with cardiovascular diseases were defined as women who had a history of at least one of following cardiovascular diseases: acyanotic CHD, cyanotic CHD, arrhythmia, cardiomyopathy, congestive heart failure (CHF), ischemic heart disease (IHD), and cardiac arrest. These disease data and medication history were screened using ICD-10 and ATC codes, respectively (S2 Table).

### Analysis

Logistic regression, and the random forest were used for the prediction of early PTB [36–42]. A random forest is a group of decision trees with a majority vote on the dependent variable. The random forest with 100 decision trees was employed in this study (100 training sets were sampled with replacements, 100 decision trees were trained with the 100 training sets, and 100 decision trees made 100 predictions). The random forest took a majority vote on the dependent variable. Data of 149,643 cases with full information were split into training and validation sets at a ratio of 80:20. Random forest feature importance was introduced for identifying major determinants of PTB and testing its associations with PM_10_ concentrate, socioeconomic status, cardiovascular disease and medication history using benzodiazepine, progesterone, and tricyclic antidepressants. Subgroup analysis of pregnant women with underlying cardiovascular diseases was performed. Major determinants were defined as variables ranked as the top 50% among all variables in the early PTB prediction model. Oversampling approach was applied so that training of machine learning could be balanced between early PTB and term birth groups. Furthermore, to determine how specific variables worked in the prediction model, SHAP (Shapley Additive Explanations) value was computed. Python (CreateSpace: Scotts Valley, 2009) was employed for the analysis between December 15, 2021 and April 15, 2022.

## Results

### Characteristics of study population

A total of 149,643 primiparous women were included in the final analysis. Among the study population, 3,066 (2.05%) women had early PTB and 10,953 (7.32%) women had at least one underlying cardiovascular disease. Maternal age at delivery was higher in women with early PTB than in those with term birth (32.19 years vs. 31.84 years, *p* < 0.0001). Most cardiovascular diseases except CHD were more common in women who had early PTB than those who had term birth. Baseline characteristics of the study population are described in Table 1. Table 2 shows monthly PM_10_ concentration data (from January 2016 to December 2016) and PM_10_ concentration of each month before delivery (from 1 to 10 months before delivery) in each group (term birth vs. early PTB). The concentration of PM_10_ was significantly different between early PTB and term birth groups in summer and early fall (from June to September). During the period from 5 to 7 months before delivery, women who had early PTB were exposed to significantly higher concentrations of PM_10_ than those who had term birth.

**Table 1:** Baseline characteristics of study population.

Variables	Term birth (n = 146,577)	Early preterm birth (n = 3,066)	P
Demographic information			
Age at delivery (years)	31.84	32.19	< 0.0001
Socioeconomic status (Insurance fee)	11.15	11.08	0.4797
Cardiovascular diseases			
Cyanotic CHD	31 (0.02%)	2 (0.07%)	0.1037
Acyanotic CHD	247 (0.17%)	6 (0.20%)	0.7169
Arrhythmia	6,327 (4.32%)	155 (5.06%)	0.0467
Cardiomyopathy	73 (0.05%)	6 (0.20%)	0.0005
Congestive heart failure	676 (0.46%)	26 (0.85%)	0.0019
Ischemic heart disease	4,078 (2.78%)	110 (3.59%)	0.0074
Cardiac arrest	7 (0.01%)	0 (0%)	0.7020
Obstetric and gynecologic diseases			
Placenta previa	489 (0.33%)	9 (0.29%)	0.7030
Threatened abortion	18,291 (12.48%)	498 (16.24%)	< 0.0001
Incompetent internal os of cervix	90 (0.06%)	4 (0.13%)	0.1309
Gestational diabetes	65,103 (44.42%)	1,444 (47.10%)	0.0031
Hypertension during pregnancy	6,164 (4.21%)	291 (9.49%)	< 0.0001
Congenital malformation of uterus	401 (0.27%)	26 (0.85%)	< 0.0001
Pelvic inflammatory disease	42,429 (28.95%)	1,085 (35.39%)	< 0.0001
Vaginitis	117,299 (80.03%)	2,515 (82.03%)	0.0060
Endometriosis	5,972 (4.07%)	213 (6.95%)	< 0.0001
Abnormal menstruation	42,370 (28.91%)	996 (32.49%)	< 0.0001
Recurrent abortion or infertility	31,572 (21.54%)	933 (30.43%)	< 0.0001
Other medical diseases			
Hypertension	17,724 (12.09%)	487 (15.88%)	< 0.0001
Diabetes	5,303 (3.62%)	193 (6.29%)	< 0.0001
Hyperlipidemia	33,098 (22.58%)	884 (28.83%)	< 0.0001
Anemia	41,169 (28.09%)	983 (32.06%)	< 0.0001
Pulmonary embolism	64 (0.04%)	1 (0.03%)	0.7714
Sepsis	84,252 (57.48%)	1,873 (61.09%)	< 0.0001
Stroke	605 (0.41%)	16 (0.52%)	0.3524
Medication			
Benzodiazepine	61,740 (42.12%)	1,480 (48.27%)	< 0.0001
Calcium channel blocker	422 (0.29%)	17 (0.55%)	0.0069
Nitrate	310 (0.21%)	5 (0.16%)	0.5627
Progesterone	23,817 (16.25%)	620 (20.22%)	< 0.0001
Hypnotic/sedative drug	7,067 (4.82%)	231 (7.53%)	< 0.0001
Tricyclic antidepressant	15,027 (10.25%)	388 (12.65%)	< 0.0001

**Table 2:** PM_10_ concentration exposed to study population.

PM_10_ concentration	Term birth (n = 146,577)	Early preterm birth (n = 3,066)	P
Monthly PM_10_ concentration (μm/m^3^)			
PM_10_ in Jan un2016	50.25	50.38	0.3898
PM_10_ in Feb 2016	47.42	47.41	0.9011
PM_10_ in Mar 2016	61.70	61.87	0.3637
PM_10_ in Apr 2016	68.18	68.35	0.3269
PM_10_ in May 2016	54.78	54.83	0.6631
PM_10_ in Jun 2016	43.22	43.48	0.0240
PM_10_ in Jul 2016	30.93	31.20	0.0024
PM_10_ in Aug 2016	34.17	34.41	0.0326
PM_10_ in Sep 2016	37.50	37.78	0.0071
PM_10_ in Oct 2016	39.40	39.58	0.1176
PM_10_ in Nov 2016	53.64	53.83	0.2232
PM_10_ in Dec 2016	48.36	48.49	0.3667
PM_10_ concentration of each month before delivery (μm/m^3^)			
PM_10_ in 10 months before delivery	47.56	45.95	< 0.0001
PM_10_ in 9 months before delivery	47.33	47.04	0.1907
PM_10_ in 8 months before delivery	46.37	47.89	< 0.0001
PM_10_ in 7 months before delivery	47.02	49.90	< 0.0001
PM_10_ in 6 months before delivery	47.11	50.05	< 0.0001
PM_10_ in 5 months before delivery	47.57	49.37	< 0.0001
PM_10_ in 4 months before delivery	47.06	47.79	0.0042
PM_10_ in 3 months before delivery	46.74	46.24	0.0459
PM_10_ in 2 months before delivery	46.11	44.40	< 0.0001
PM_10_ in 1 month before delivery	45.30	43.27	< 0.0001

### Prediction model for early PTB and effect of PM_10_ on PTB

Table 3(a) presents accuracy, sensitivity, specificity and areas under the operating-characteristic-curve (AUC) of the early PTB prediction model. With the random forest model for oversampled data, the AUC was 0.9988 and the accuracy was 0.9984. With the logistic-regression model, the AUC was 0.6787 and the accuracy was 0.5450. The performance of the random forest model was superior to the logistic regression model. The model with oversampled data showed greater AUC than that model with the original data. Therefore, we considered findings of logistic regression as supplementary findings.

**Table 3:** Performance measures of prediction model. **(a)** Prediction model for early PTB in total study population, **(b-1)** Prediction model for early PTB in women with underlying cardiovascular diseases, **(b-2)** Prediction model for early PTB in women without underlying cardiovascular diseases.

		Original data	Oversampled data
**(a)**			
**Logistic Regression**	**Accuracy**	0.9805	0.6787
	**Sensitivity**	0.0000	0.5508
	**Specificity**	0.9805	0.6914
	**AUC**	0.5000	0.5450
**Random Forest**	**Accuracy**	0.9803	0.9984
	**Sensitivity**	0.0000	0.9951
	**Specificity**	0.9805	1.0000
	**AUC**	0.4999	0.9988
**(b-1)**			
**Logistic Regression**	**Accuracy**	0.9759	0.6683
	**Sensitivity**	0.0000	0.5109
	**Specificity**	0.9759	0.6924
	**AUC**	0.5000	0.5527
**Random Forest**	**Accuracy**	0.9749	0.9981
	**Sensitivity**	0.0000	0.9942
	**Specificity**	0.9759	1.0000
	**AUC**	0.4995	0.9985
**(b-2)**			
**Logistic Regression**	**Accuracy**	0.9817	0.6822
	**Sensitivity**	0.0000	0.5737
	**Specificity**	0.9817	0.6923
	**AUC**	0.5000	0.5465
**Random Forest**	**Accuracy**	0.9816	0.9985
	**Sensitivity**	0.0000	0.9954
	**Specificity**	0.9817	1.0000
	**AUC**	0.4999	0.9988

Abbreviation: AUC, Areas under the operating-characteristic-curve.

**Fig 1:**
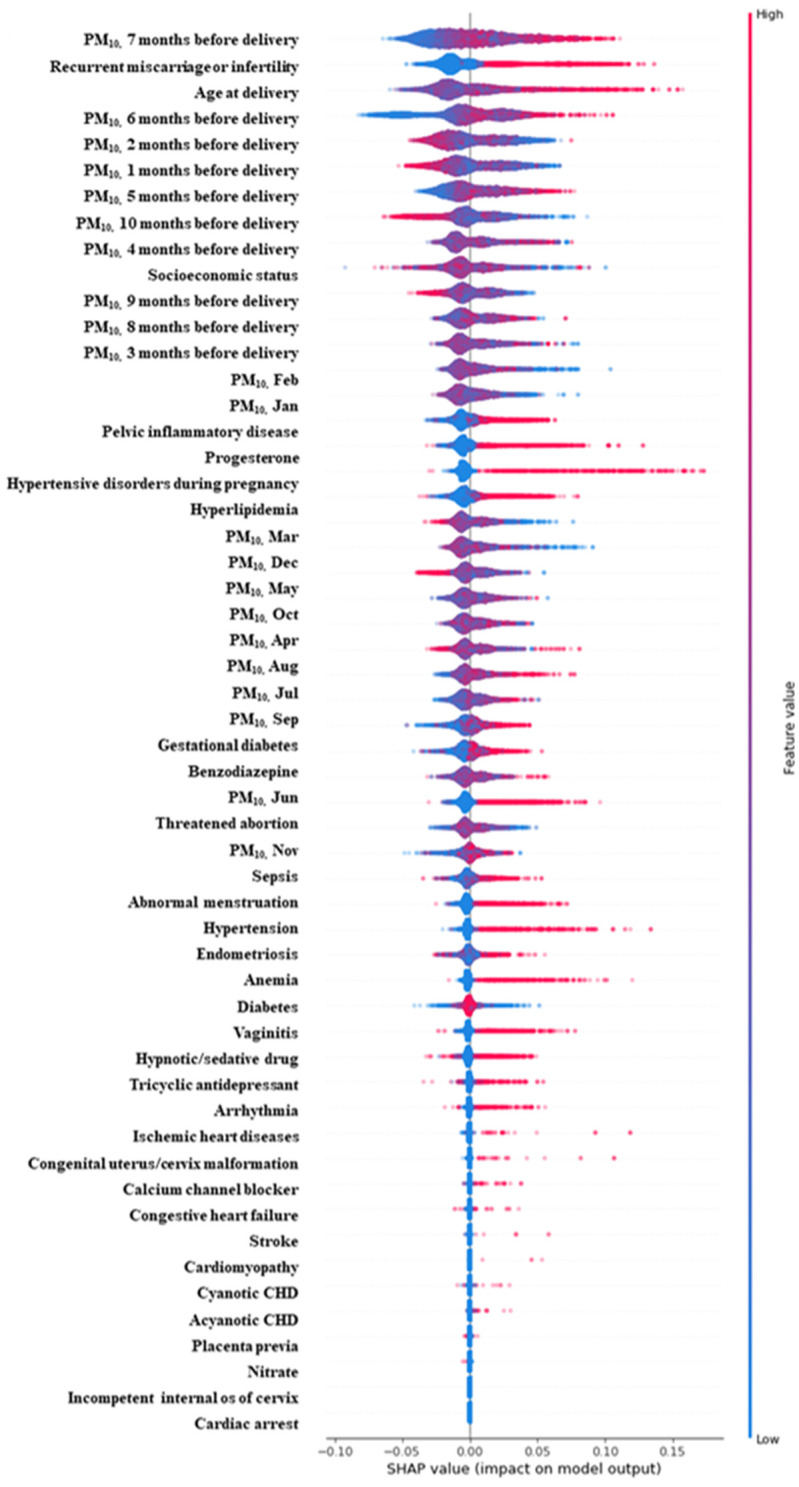
SHAP value of early-PTB prediction model.

Results of feature importance of major determinants of early PTB are presented in Table 4. It should be noted that most of the major determinants of early PTB for oversampling data were similar to those for original data. Socioeconomic status influenced PTB the most, followed by age at delivery. Among 27 major determinants of early PTB, PM_10_ concentration of each specific month before delivery ranked within top-10 major determinants of early PTB in oversampled data. PM_10_ concentration of each period before delivery (i.e., PM_10_ concentrations of five months before delivery) had more impact on early PTB than PM_10_ concentration of a specific month (i.e., PM_10_ concentration of December). This trend was also shown in the original data. This finding implies that maternal exposure to PM_10_ is associated with early PTB and that the impact of PM_10_ is greater than well-known contributing factors of early PTB, such as infection (feature importance in oversampled data, PM_10_ concentration in six months before delivery (0.0320) vs. pelvic inflammatory disease (0.0198) vs. vaginitis (0.0197)) (Table 4(a)). Fig 1 presents SHAP value of the prediction model which shows the sign and magnitude for the effect of a major determinant on early PTB. SHAP value of PM_10_ concentration of 5 to 7 months before delivery (first and early second trimester of pregnancy) ranked high. Higher PM_10_ concentration increased the risk of early PTB. The probability of early PTB was increased by 7.73%, 10.58% or 11.11% if a variable PM_10_ concentration of 5, 6, or 7 months before delivery was included to the prediction model.

**Table 4:** Random forest feature importance of prediction model for early PTB (top 27 variables). **(a)** Feature importance in total study population, **(b-1)** Prediction model for early PTB in women with underlying cardiovascular diseases (original data), **(b-2)** Prediction model for early PTB in women with underlying cardiovascular diseases (oversampled data).

**(a)**				
	**Original data**		**Oversampled data**	
**Rank**	**Variables**	**Feature importance**	**Variables**	**Feature importance**
1	Socioeconomic status	0.1392	Socioeconomic status	0.1017
2	Age at delivery	0.1273	Age at delivery	0.0928
3	Gestational diabetes	0.0367	PM_10_, 6 months before delivery	0.0320
4	Sepsis	0.0349	PM_10_, 2 months before delivery	0.0313
5	Benzodiazepine	0.0348	PM_10_, 7 months before delivery	0.0302
6	Abnormal menstruation	0.0323	PM_10_, 1 month before delivery	0.0298
7	Anemia	0.0295	PM_10_, 10 months before delivery	0.0290
8	Pelvic inflammatory disease	0.0286	PM_10_, 4 months before delivery	0.0283
9	Vaginitis	0.0253	PM_10_, 9 months before delivery	0.0280
10	Hyperlipidemia	0.0209	PM_10_, 3 months before delivery	0.0277
11	Progesterone	0.0208	PM_10_, 5 months before delivery	0.0277
12	PM_10_, 1 month before delivery	0.0203	Gestational diabetes	0.0274
13	PM_10_, 2 months before delivery	0.0202	PM_10_, 8 months before delivery	0.0269
14	PM_10_, 5 months before delivery	0.0199	Sepsis	0.0257
15	PM_10_, 4 months before delivery	0.0198	Benzodiazepine	0.0236
16	PM_10_, 3 months before delivery	0.0197	Abnormal menstruation	0.0229
17	PM_10_, 8 months before delivery	0.0196	Anemia	0.0219
18	PM_10_, 6 months before delivery	0.0195	Pelvic inflammatory disease	0.0198
19	PM_10_, 10 months before delivery	0.0194	Vaginitis	0.0197
20	PM_10_, 7 months before delivery	0.0193	PM_10_, Apr	0.0193
21	PM_10_, 9 months before delivery	0.0191	PM_10_, Jan	0.0188
22	Miscarriage or infertility	0.0184	PM_10_, Mar	0.0188
23	TA	0.0178	PM_10_, May	0.0186
24	Hypertension	0.0157	PM_10_, Jul	0.0185
25	Tricyclic antidepressant	0.0152	PM_10_, Aug	0.0185
26	PM_10_, Feb	0.0129	PM_10_, Feb	0.0184
27	PM_10_, Mar	0.0123	PM_10_, Jun	0.0183
**(b-1)**				
	**Women with cardiovascular diseases**		**Women without cardiovascular diseases**	
**Rank**	**Variables**	**Feature importance**	**Variables**	**Feature importance**
1	Socioeconomic status	0.0793	Socioeconomic status	0.1490
2	Age at delivery	0.0789	Age at delivery	0.1347
3	PM_10_, 1 month before delivery	0.0353	Gestational diabetes	0.0391
4	PM_10_, 3 months before delivery	0.0344	Sepsis	0.0357
5	PM_10_, 2 months before delivery	0.0342	Benzodiazepine	0.0337
6	PM_10_, 4 months before delivery	0.0318	Abnormal menstruation	0.0330
7	PM_10_, 6 months before delivery	0.0315	Anemia	0.0319
8	PM_10_, 5 months before delivery	0.0305	Pelvic inflammatory disease	0.0296
9	PM_10_, 7 months before delivery	0.0304	Vaginitis	0.0263
10	PM_10_, 10 months before delivery	0.0297	Hyperlipidemia	0.0258
11	PM_10_, 9 months before delivery	0.0292	Progesterone	0.0235
12	PM_10_, 8 months before delivery	0.0278	PM_10_, 1 month before delivery	0.0201
13	Anemia	0.0222	PM_10_, 2 months before delivery	0.0196
14	Benzodiazepine	0.0220	PM_10_, 5 months before delivery	0.0195
15	Recurrent miscarriage or infertility	0.0216	Threatened abortion	0.0193
16	PM_10_, Dec	0.0209	PM_10_, 7 months before delivery	0.0193
17	Gestational diabetes	0.0209	PM_10_, 8 months before delivery	0.0193
18	Hyperlipidemia	0.0207	PM_10_, 4 months before delivery	0.0191
19	Pelvic inflammatory disease	0.0202	PM_10_, 6 months before delivery	0.0191
20	PM_10_, Jun	0.0199	PM_10_, 3 months before delivery	0.0191
21	PM_10_, Apr	0.0198	PM_10_, 9 months before delivery	0.0186
22	PM_10_, Sep	0.0198	PM_10_, 10 months before delivery	0.0183
23	PM_10_, Aug	0.0197	Hypertension	0.0161
24	Sepsis	0.0193	Recurrent miscarriage or infertility	0.0159
25	PM_10_, Feb	0.0188	Tricyclic antidepressant	0.0159
26	Abnormal menstruation	0.0188	PM_10_, Apr	0.0118
27	PM_10_, Jul	0.0185	PM_10_, Jan	0.0117
**(b-2)**				
	**Women with cardiovascular diseases**		**Women without cardiovascular diseases**	
**Rank**	**Variables**	**Feature importance**	**Variables**	**Feature importance**
1	Socioeconomic status	0.0581	Socioeconomic status	0.1052
2	Age at delivery	0.0565	Age at delivery	0.0966
3	PM_10_, 2 months before delivery	0.0462	PM_10_, 6 months before delivery	0.0327
4	PM_10_, 10 months before delivery	0.0454	PM_10_, 2 months before delivery	0.0306
5	PM_10_, 7 months before delivery	0.0398	PM_10_, 7 months before delivery	0.0303
6	PM_10_, 6 months before delivery	0.0392	PM_10_, 1 month before delivery	0.0301
7	PM_10_, 4 months before delivery	0.0381	PM_10_, 10 months before delivery	0.0294
8	PM_10_, 9 months before delivery	0.0371	PM_10_, 8 months before delivery	0.0283
9	PM_10_, 3 months before delivery	0.0354	PM_10_, 9 months before delivery	0.0282
10	PM_10_, 1 month before delivery	0.0349	PM_10_, 4 months before delivery	0.0282
11	PM_10_, 5 months before delivery	0.0345	PM_10_, 3 months before delivery	0.0281
12	PM_10_, 8 months before delivery	0.0338	PM_10_, 5 months before delivery	0.0277
13	PM_10_, Jan	0.0294	Gestational diabetes	0.0272
14	PM_10_, Dec	0.0256	Sepsis	0.0262
15	PM_10_, Aug	0.0253	Benzodiazepine	0.0237
16	PM_10_, Jul	0.0250	Abnormal menstruation	0.0232
17	PM_10_, Sep	0.0248	Anemia	0.0228
18	PM_10_, Nov	0.0243	Pelvic inflammatory disease	0.0201
19	PM_10_, Apr	0.0242	Vaginitis	0.0201
20	PM_10_, May	0.0241	PM_10_, Jan	0.0196
21	PM_10_, Mar	0.0239	PM_10_, Apr	0.0195
22	PM_10_, Feb	0.0236	PM_10_, Feb	0.0188
23	PM_10_, Oct	0.0230	PM_10_, Mar	0.0187
24	PM_10_, Jun	0.0229	PM_10_, Dec	0.0186
25	Benzodiazepine	0.0155	PM_10_, Jul	0.0184
26	Sepsis	0.0142	PM_10_, May	0.0183
27	Hyperlipidemia	0.0141	Hyperlipidemia	0.0182

### Effect of PM_10_ on PTB in women with underlying cardiovascular diseases

Subgroup analysis of women with underlying cardiovascular diseases was conducted. Table 3(b) presents accuracy, sensitivity, specificity and AUC of the subgroup analysis. Early PTB prediction model by random forest of oversampled data in both women with and without cardiovascular diseases also showed a fine performance. Table 4(b) presents feature importance of major determinants of early PTB in subgroup analysis. A total of 22 variables of PM_10_ concentration ranked in 3^rd^ to 24^th^ of feature importance in women with cardiovascular diseases. However, 17 variables of PM_10_ concentration were ranked as major determinants in women without cardiovascular diseases. The rank of PM_10_ concentration was relatively lower in women without cardiovascular diseases than in those with cardiovascular diseases. This implies that women with cardiovascular diseases might be more susceptible to PM_10_ concentration in terms of risk for early PTB than those without cardiovascular diseases. This trend was also observed in original data in a stronger way.

## Discussion

### Main finding

This large population-based cohort study set the prediction model for early PTB using random forest. The AUC and accuracy of PTB prediction model using random forest were 0.9988 and 0.9984, respectively. We found that PM_10_ concentration of each period before delivery was a major contributor to early PTB. We also found that the higher PM_10_ concentration of 5 to 7 months before delivery increased the risk of early PTB based on the SHAP value. Furthermore, women with cardiovascular diseases were found to be more vulnerable to PM_10_ concentration than those without cardiovascular diseases.

### Effects of PM_10_ on PTB

Although the pathophysiology of PM_10_ on PTB has not yet been clearly demonstrated, PM_10_ induced inflammation and oxidative stress are considered as key pathway of PM_10_ causing PTB [39–44]. In addition, because PM concentration has seasonal difference which might have different effects on PTB depending on the period of exposure, some studies have analyzed the effect of PM on PTB according to the trimester of pregnancy [18–22]. Considering these, we analyzed the effect of PM_10_ on early PTB according to the concentration of each period before delivery and the specific month which could reflect the season. The current study found that maternal exposure to PM_10_ according to the period of pregnancy (PM_10_ concentration of each month before delivery) was more associated with the risk of early PTB than the concentration of PM_10_ itself (monthly PM_10_ concentration). In addition, higher PM_10_ concentration in 5 to 7 months before delivery (the first and early second trimester) was a major contributor to early PTB and associated with an increased risk of PTB. This result was consistent with previous studies showing that maternal exposure to PM_10_ in first and second trimesters could significantly increase the risk of PTB [18–22]. Throughout the current study, we assumed that maternal exposure to PM_10_ during the first and early second trimester of pregnancy might have more critical effects on PTB compared to the exposure during other periods.

### Effects of PM_10_ on PTB in women with cardiovascular diseases

The pathological mechanism of PM for cardiovascular diseases can be broadly divided into direct translocation and indirect pathway [45]. Direct action has a direct effect on the cardiovascular system as ultrafine particles translocates through the blood stream [45]. The indirect pathway affects cardiovascular diseases by oxidative stress and activation of the inflammation pathway [45]. Several studies have reported that pro-inflammatory cytokines are increased in subjects exposed to PM [46–48]. Systemic inflammatory response can promote atherosclerosis, coagulability, and endothelial dysfunction, which ultimately affects the cardiovascular system [43]. In addition, PM can stimulate the autonomic nervous system and the hypothalamic-pituitary-adrenal (HPA) axis. It is also associated with systemic inflammatory responses and atherosclerosis [49–54]. Women with cardiovascular diseases have suboptimal cardiac adaptation during pregnancy compared to healthy women. They also have more underlying cardiovascular risk factors that can increase the risk of PTB, which will increase the likelihood of PTB [31, 55–59]. In this study, we found that PM_10_ had a relatively stronger effect on early PTB of pregnant women with cardiovascular diseases than those without cardiovascular diseases. We assumed that PM_10_ exacerbate the cardiovascular function of pregnant women with underlying cardiovascular diseases, and this can further increase the risk of early PTB.

### Strength and limitation

The strength of the current study was that we used large-scale population-based data and analyzed these data with machine learning, one of the optimal methods for analyzing large amounts of data. Moreover, we used various variables including demographic/socioeconomic, obstetric, and gynecologic, cardiovascular, and other medical information as confounding factors. Furthermore, we analyzed the timing and co-morbidities that might exaggerate the effect of PM_10_ on early PTB. However, this study also has some limitations. First, we could not present the actual gestational age at delivery because we used original data from KNHIS claims that only provided ICD-10 code, not the actual gestational age at delivery. In addition, we could not subdivide the cause of early PTB. There are various mechanisms of early PTB including spontaneous preterm labor, severe maternal morbidity such as preeclampsia, and severe fetal morbidity such as non-reassuring fetal heart rate. However, we could not analyze the mechanism of PTB due to the lack of information in the original data. Lastly, other air pollutants such as PM_2.5_, NO_2_, and O_3_ were not evaluated.

## Conclusion

With this large population-based cohort study using machine learning, we found that maternal exposure to PM_10_ was a major contributor to early PTB. Moreover, we found that in the context of PTB, pregnant women with cardiovascular diseases are a high-risk group of PM_10_ and the first and early second trimester is a high-risk period of PM_10_. The current study emphasized the importance of PM_10_ as an overlooked risk factor for PTB. We believe that these findings can alert the risk of PM_10_ to both obstetricians and pregnant women, and the effort to reduce the maternal exposure to PM_10_, especially in pregnant women with cardiovascular diseases in their first and early second trimester is needed.

## Supporting information

S1 Table ICD-10 Codes and procedure codes for preterm birth and cardiovascular diseases. (DOC)

S2 Table ATC codes for medications. (DOC)
