# Supplementary material for: Association between early preterm birth and maternal exposure to fine particular matter (PM10): A nation-wide population-based cohort study using machine learning
Source: PLoS One. 2023 Aug 7;18(8):e0289486. doi: 10.1371/journal.pone.0289486 (PMC10406328; doi:10.1371/journal.pone.0289486)
Supplement: S1 Table — (DOC) [file pone.0289486.s001.doc]

**Table S1**. ICD-10 Codes and procedure codes for preterm birth and cardiovascular diseases

| Variables | Code | Description |
| --- | --- | --- |
| Early PTB | O42.00 | Premature rupture of membranes, onset of labor within 24 hours (0-33 weeks of gestation) |
|  | O42.10 | Premature rupture of membranes, onset of labor after 24 hours (0-33 weeks of gestation) |
|  | O42.20 | Premature rupture of membranes, labor delayed by therapy (0-33 weeks of gestation) |
|  | O42.90 | Premature rupture of membranes, unspecified (0-33 weeks of gestation) |
|  | O60.10 | Preterm spontaneous labor with preterm delivery (0-21 weeks of gestation) |
|  | O60.11 | Preterm spontaneous labor with preterm delivery (22-33 weeks of gestation) |
|  | O60.30 | Preterm delivery without spontaneous labor (0-21 weeks of gestation) |
|  | O60.31 | Preterm delivery without spontaneous labor (22-33 weeks of gestation) |
| Pelvic inflammatory disease | N73 | Other female pelvic inflammatory diseases |
|  | N74 | Female pelvic inflammatory disorders in diseases classified elsewhere |
| Vaginitis | N76 | Other inflammation of vagina and vulva |
| Endometriosis | N80 | Endometriosis |
| Abnormal menstruation | N91.4 | Secondary oligomenorrhoea |
|  | N91.5 | Oligomenorrhoea, unspecified |
|  | N92.0 | Excessive and frequent menstruation with regular cycle |
|  | N92.1 | Excessive and frequent menstruation with irregular cycle |
|  | N92.3 | Ovulation bleeding |
|  | N92.4 | Excessive bleeding in the premenopausal period |
|  | N92.5 | Other specified irregular menstruation |
|  | N92.6 | Irregular menstruation, unspecified |
| Recurrent abortion or infertility | N96 | Habitual aborter |
|  | N97 | Female infertility |
|  | N98 | Complications associated with artificial fertilization |
| Congenital uterus/cervix malformation | Q51 | Congenital malformations of uterus and cervix |
| Gestational diabetes | O24.4 | Diabetes mellitus arising in pregnancy |
|  | O24.9 | Diabetes mellitus in pregnancy, unspecified |
| Hypertension during pregnancy | O13 | Gestational [pregnancy-induced] hypertension |
|  | O14 | Pre-eclampsia |
|  | O15 | Eclampsia |
|  | O16 | Unspecified maternal hypertension |
| Previa | O44 | Placenta praevia |
| TA | O20.0 | Threatened abortion |
| IIOC | N88.3 | Incompetence of cervix uteri |
| Acyanotic CHD | Q21.0 | Ventricular septal defect |
|  | Q21.1 | Atrial septal defect |
|  | Q21.2 | Atrioventricular septal defect |
|  | Q21.4 | Aortopulmonary septal defect |
|  | Q21.9 | Congenital malformation of cardiac septum, unspecified |
|  | Q22.1 | Congenital pulmonary valve stenosis |
|  | Q22.2 | Congenital pulmonary valve insufficiency |
|  | Q22.3 | Other congenital malformations of pulmonary valve |
|  | Q24.3 | Pulmonary infundibular stenosis |
|  | Q25.6 | Stenosis of pulmonary artery |
|  | Q25.7 | Other congenital malformations of pulmonary artery |
|  | Q22.8 | Other congenital malformations of tricuspid valve |
|  | Q22.9 | Congenital malformation of tricuspid valve, unspecified |
|  | Q23.0 | Congenital stenosis of aortic valve |
|  | Q23.1 | Congenital insufficiency of aortic valve |
|  | Q24.4 | Congenital subaortic stenosis |
|  | Q25.1 | Coarctation of aorta |
|  | Q25.2 | Atresia of aorta |
|  | Q25.3 | Stenosis of aorta |
|  | Q25.4 | Other congenital malformations of aorta |
|  | Q23.2 | Congenital mitral stenosis |
|  | Q23.3 | Congenital mitral insufficiency |
|  | Q23.8 | Other congenital malformations of aortic and mitral valves |
|  | Q23.9 | Congenital malformation of aortic and mitral valves, unspecified |
|  | Q24.2 | Cor triatriatum |
|  | Q24.5 | Malformation of coronary vessels |
|  | Q25.0 | Patent ductus arteriosus |
|  | Q25.6 | Stenosis of pulmonary artery |
|  | Q25.7 | Other congenital malformations of pulmonary artery |
|  | Q25.8 | Other congenital malformations of great arteries |
|  | Q25.9 | Congenital malformation of great arteries, unspecified |
|  | Q26.3 | Partial anomalous pulmonary venous connection |
|  | Q26.4 | Anomalous pulmonary venous connection, unspecified |
| Cyanotic CHD | Q20.0 | Common arterial trunk |
|  | Q20.1 | Double outlet right ventricle |
|  | Q20.2 | Double outlet left ventricle |
|  | Q20.3 | Discordant ventriculoarterial connection |
|  | Q20.4 | Double inlet ventricle |
|  | Q20.5 | Discordant atrioventricular connection |
|  | Q21.3 | Tetralogy of Fallot |
|  | Q21.8 | Other congenital malformations of cardiac septa |
|  | I27.8 | Other specified pulmonary heart diseases |
|  | Q22.0 | Pulmonary valve atresia |
|  | Q22.4 | Congenital tricuspid stenosis, atresia |
|  | Q22.6 | Hypoplastic right heart syndrome |
|  | Q25.5 | Atresia of pulmonary artery |
|  | Q22.5 | Ebstein anomaly |
|  | Q23.4 | Hypoplastic left heart syndrome |
|  | Q26.0 | Congenital stenosis of vena cava |
|  | Q26.8 | Other congenital malformations of great veins |
|  | Q26.9 | Congenital malformation of great vein, unspecified |
|  | Q26.2 | Total anomalous pulmonary venous connection |
| Arrhythmia | I44 | Atrioventricular and left bundle-branch block |
|  | I45 | Other conduction disorders |
|  | I47 | Paroxysmal tachycardia |
|  | I48 | Atrial fibrillation and flutter |
|  | I49 | Other cardiac arrhythmias |
|  | R00.1 | Bradycardia, unspecified |
| Cardiac arrest | I46 | Cardiac arrest |
| Cardiomyopathy | I42 | Cardiomyopathy |
| Congestive heart failure | I11.0 | Hypertensive heart disease with (congestive) heart failure |
|  | I13.0 | Hypertensive heart and renal disease with (congestive) heart failure |
|  | I13.2 | Hypertensive heart and renal disease with both (congestive) heart failure and renal failure |
|  | I50 | Heart failure |
| Hyperlipidemia | E78 | Disorders of lipoprotein metabolism and other lipidemias |
| Ischemic heart disease | I20 | Angina pectoris |
|  | I21 | Acute myocardial infarction |
|  | I22 | Subsequent myocardial infarction |
|  | I23 | Certain current complications following acute myocardial infarction |
|  | I24 | Other acute ischemic heart diseases |
|  | I25 | Chronic ischemic heart disease |
| Pulmonary embolism | I26 | Pulmonary embolism |
|  | O88.2 | Obstetric (pulmonary) embolism |
|  | O08.2 | Embolism following abortion and ectopic and molar pregnancy |
|  | O03.2 | Incomplete spontaneous abortion, complicated by embolism |
|  | O03.7 | Complete or unspecified spontaneous abortion, complicated by embolism |
|  | O06.2 | Incomplete unspecified abortion, complicated by embolism |
|  | O05.2 | Incomplete other abortion, complicated by embolism |
|  | O07.7 | Other and unspecified failed attempted abortion, complicated by embolism |
|  | O04.2 | Incomplete medical abortion, complicated by embolism |
|  | O04.7 | Complete or unspecified medical abortion, complicated by embolism |
|  | O05.7 | Complete or unspecified other abortion, complicated by embolism |
|  | O88 | Obstetric embolism |
| Sepsis | A40 | Streptococcal sepsis |
|  | A41 | Other sepsis |
|  | A42 | Actinomycosis |
|  | A32 | Listeriosis |
|  | B37 | Candidiasis |
|  | O85 | Puerperal sepsis |
| Stroke | I60 | Subarachnoid hemorrhage |
|  | I61 | Intracerebral hemorrhage |
|  | I62 | Other nontraumatic intracranial hemorrhage |
|  | I63 | Cerebral infarction |
| Diabetes | E10 | Type 1 diabetes mellitus |
|  | E11 | Type 2 diabetes mellitus |
|  | E12 | Malnutrition-related diabetes mellitus |
|  | E13 | Other specified diabetes mellitus |
|  | E14 | Unspecified diabetes mellitus |
|  | O24.0 | Pre-existing type 1 diabetes mellitus |
|  | O24.1 | Pre-existing type 2 diabetes mellitus |
|  | O24.2 | Pre-existing malnutrition-related diabetes mellitus |
|  | O24.3 | Pre-existing diabetes mellitus, unspecified |
| Hypertension | I10 | Essential(primary) hypertension |
|  | I11 | Hypertensive heart disease |
|  | I12 | Hypertensive renal disease |
|  | I13 | Hypertensive heart and renal disease |
|  | I15 | Secondary hypertension |
|  | O10 | Pre-existing hypertension complicating pregnancy, childbirth and the puerperium |
|  | O11 | Pre-eclampsia superimposed on chronic hypertension |
| Anemia | D50 | Iron deficiency anemia |
|  | D51 | Vitamin B12 deficiency anemia |
|  | D52 | Folate deficiency anemia |
|  | D53 | Other nutritional anemias |
|  | D55 | Anemia due to enzyme disorders |
|  | D57 | Sickle-cell disorders |
|  | D58 | Other hereditary hemolytic anemias |
|  | D59 | Acquired hemolytic anemia |
|  | D60 | Acquired pure red cell aplasia |
|  | D61 | Other aplastic anemias and other bone marrow failure syndromes |
|  | D62 | Acute posthemorrhagic anemia |
|  | D63 | Anemia in chronic diseases classified elsewhere |
|  | D64 | Other anemias |

PTB indicates preterm birth; PPROM, preterm premature rupture of membranes; PTL, preterm labor; WPW, Wolff-Parkinson-White syndrome; SVT, supraventricular tachycardia; AF, atrial fibrillation; AFL, atrial flutter; VA, ventricular arrhythmia; SSS, sick sinus syndrome.
