# Supplementary material for: Association between early preterm birth and maternal exposure to fine particular matter (PM10): A nation-wide population-based cohort study using machine learning
Source: PLoS One. 2023 Aug 7;18(8):e0289486. doi: 10.1371/journal.pone.0289486 (PMC10406328; doi:10.1371/journal.pone.0289486)
Supplement: S2 Table — (DOC) [file pone.0289486.s002.doc]

**Table S2**. ATC Codes for Medications

| **Code** | **Medication** |
| --- | --- |
| N05BA | Benzodiazepine |
| N05CD |
| N05CF |
| C08 | Calcium channel blocker |
| C01DA | Nitrate |
| G03 | Progesterone |
| N05C | Hypnotics and sedatives |
| N06A | Tricyclic antidepressant |

ATC indicates Anatomical Therapeutic Chemical
